# Prioritizing Equity in Design and Implementation of Consumer-Facing Digital Resources to Support Engagement and Shared Decision-Making

**DOI:** 10.2196/92842

**Published:** 2026-08-11

**Authors:** Jenna Smith, Julie Ayre, Carissa Bonner, Danielle Muscat, Heather L Shepherd, Eva Hussain, Husna Amani, Marguerite Tracy, Kristie R Weir, Kathleen McFadden, Kirsten J McCaffery, Jolyn Hersch

**Affiliations:** 1 Sydney Health Literacy Lab, Sydney School of Public Health, Faculty of Medicine and Health, The University of Sydney, Sydney, New South Wales, 2006, Australia, 61 286270095; 2 Leeder Centre for Health Policy, Economics & Data, Sydney School of Public Health, Faculty of Medicine and Health, The University of Sydney, Sydney, New South Wales, Australia; 3 Sydney Health Literacy Lab Consumer Panel (Co-SHeLL), Sydney School of Public Health, Faculty of Medicine and Health, The University of Sydney, Sydney, New South Wales, Australia; 4 Community member and disability advocate, Melbourne, Victoria, Australia; 5 General Practice Clinical School, Sydney Medical School, Faculty of Medicine and Health, The University of Sydney, Sydney, New South Wales, Australia; 6 Institute of Primary Health Care (BIHAM), University of Bern, Bern, Switzerland

**Keywords:** digital health, shared decision-making, equity, patient decision aids, health literacy

## Abstract

A digitally enabled health system offers the opportunity to address gaps in the implementation of shared decision-making, a collaborative process between health professionals and consumers to decide on the best test, treatment, or management option based on clinical evidence and the consumer’s values and informed preferences. There is increasing design and availability of digital tools online to support shared decision-making. Providing opportunities for all consumers to make shared health care decisions is not only key to achieving safer, higher-quality health care, but also an ethical and social imperative that upholds the fundamental rights of every health care consumer. However, there is a risk that digital tools intended to support shared decision-making might worsen inequities in access to and use of health information. In this Viewpoint, we use the case study of digital decision aids to argue that equity should be proactively prioritized as these tools are increasingly designed, accessed, and adopted. We highlight the need for digital health researchers and organizations to (1) address health literacy and accessibility by design, (2) meaningfully co-design tools with underrepresented end users, and (3) generate evidence among underrepresented end users on how emerging digital decision aid features with potential for meeting more diverse needs (eg, interactivity, tailoring, and AI) impact decision-making outcomes. We summarize the current gaps in addressing these recommendations in the development of digital decision aids. Regarding implementation, we also urge policymakers advocating for consumer engagement in health decisions to invest in (1) national, standardized online portals that streamline and enhance consumer and clinician access to co-designed digital decision aids that meet the needs of users with varying levels of health literacy and (2) digital inclusion initiatives that ensure equitable access and adoption of digital tools to support shared decision-making.

## Introduction

The rise of digital health and advocacy for patient engagement in health decisions holds promise for the implementation of resources to support shared decision-making. Shared decision-making is a process where health consumers and carers are supported to consider evidence-based information alongside their own personal goals and values when making health decisions with health care providers [1]. This process leads to more informed health decisions and greater patient engagement [2], outcomes that are increasingly embedded in international digital health policies and strategies, as well as clinical care guidelines (eg, the National Institute for Health and Care Excellence [NICE] and the Australian Commission on Safety and Quality in Health Care). Supporting opportunities for all consumers to engage in decision-making about their health is key to achieving safer, higher-quality health care that suits individual needs. Furthermore, it is an ethical and social imperative respecting the fundamental rights of health consumers.

Patient decision aids are a common example of various decision support tools that have been developed over the last 20 years. Traditionally designed in paper formats such as flyers or booklets, they state the test, treatment, or management options available, outline the benefits and harms for each option, and help consumers clarify what matters most to them [2]. Decision aids may be standalone tools for consumers to use outside a clinical encounter, before a clinical encounter, and/or to supplement the decision-making process during and after the encounter. Decades of research demonstrate the effectiveness of decision aids in supporting shared decision-making through increased consumer knowledge and more active roles in decisions, as well as more accurate risk perceptions among both clinicians and consumers [2].

With increasingly digitalized health systems, the landscape of decision aid development has also followed. Digital decision aids are now either simply being adapted from paper-based origins for online availability or designed digitally from the outset [3]. There is now a vast international landscape of decision aids freely available online, with 30% available in the Netherlands and 20% in the United States [4]. Interactive versions are also beginning to be developed for more user engagement and a more dynamic decision process between consumers and clinicians (eg, allowing consumers to input questions in advance to facilitate discussion during clinical encounters or easily reference recorded notes if desired when using the tool after an encounter) [5].

Health systems are also beginning to integrate digital decision aids into existing digital platforms and environments, with promise for addressing the historical and continuing challenge of implementing decision aids in practice. Harnessing digital innovations for more responsive and sustainable shared decision-making also continues to be recognized as a priority for research and implementation by the international shared decision-making community [6]. This promise is reflected in global examples of health care systems beginning to realize the potential of decision aids. For instance, Brazil aims to incorporate decision aids in their mobile patient record app in 2026, Meu SUS Digital, allowing health consumers to monitor their health information and clinical history and access continuity of care [7]. Another example is the United Kingdom’s NICE decision aid library of 29 tools, part of a wider effort to implement their shared decision-making guidelines [8]. Germany, Taiwan, Denmark, and the United States have also integrated shared decision-making processes in hospitals to various degrees (bolstered through web-based decision aids, health professional education, and health insurer support) [9-13].

Such strategies to increase access and integration are promising for addressing well-documented shared decision-making implementation barriers. However, opportunistic digitalization strategies that focus on accessing decision aids alone or do not consider diverse needs when leveraging interactive features risk perpetuating existing and protracted inequities in development and implementation at the levels of research, implementation, and policy. Moreover, they fail to understand and begin to address the even more complex barriers to the adoption of digital decision aids among underrepresented groups. For example, in the Netherlands, where shared decision-making implementation outpaces that of other high-income countries due to strong ministerial support and funding, decision aids accessed online and through the Patient Federation lack understandability and the use of risk communication strategies for people with lower health literacy and are not developed together with such users [14].

The primary aim of this Viewpoint is to highlight how the promise of digitally designed consumer-facing resources can be realized to support more equitable shared decision-making research and practice. Using the case study of digital decision aids with an emerging evidence base, we first make 3 recommendations for how this promise can be realized through design to suit the diverse needs of end users. We then advocate for national initiatives that prioritize equity in the implementation of digital decision aids, especially in health systems that embed consumer engagement and shared decision-making in clinical care guidelines. Although we focus on digital decision aids, our recommendations also extend to the design and implementation of other consumer-facing digital resources intended to support shared decision-making (eg, question prompt lists, information sheets, and videos).

## Prioritizing Equity in Digital Decision Aid Design

Limited attention to equity in efforts to support access to health information is a real and well-evidenced problem for the evolving fields of digital health and shared decision-making [15-17]. Shared decision-making research typically focuses on the needs of those who are highly educated, fluent in the local dominant language, high in digital literacy, and already motivated to engage [18,19]. Only 11% of decision aids in a Cochrane review of randomized controlled trials were developed for, or evaluated with, consumers who are socially disadvantaged with respect to health literacy, education, income, race and ethnicity, neighborhood, or health insurance [15], despite these groups being at higher risk of poor health outcomes and representing much larger proportions of consumers in health systems who could benefit from decision support. Consumers experiencing inequities based on ability are also more likely to face a range of unique barriers in accessing and understanding health information and could greatly benefit from decision support [20] yet are not well represented in the large body of decision aid research. There is also limited understanding of intersectionality, the process by which multiple social identities intersect to shape individuals’ experiences. For example, older adults have been included in decision aid research, with limited attention to those more vulnerable due to intersecting inequities (eg, lower health literacy or education) [21].

To embed the prioritization of equity through inclusion and accessibility in routine processes of digital decision aid design, there is a need to (1) address health literacy and accessibility by design, (2) meaningfully co-design tools with typically underrepresented end users, and (3) generate evidence among underrepresented end users on how emerging digital features with potential for meeting more diverse needs (eg, interactivity, tailoring, and AI) impact decision-making outcomes.

### Addressing Health Literacy and Accessibility by Design

The longstanding issues for decision aids outlined above seem to have persisted into digital formats now available online. For example, systematic reviews of digital decision aids for cardiovascular disease prevention found that they were not designed to meet the needs of people with lower literacy, with inaccessible aspects including high-grade reading levels [22]. This limited consideration of a foundational health literacy principle restricts efforts to strengthen and sustain patient engagement through shared decision-making [23]. Furthermore, it misses an opportunity to increase equitable engagement when we know that both consumers and clinicians respond positively to health-literate decision aid design [24]. When decision aids are explicitly designed for or evaluated among groups with low health literacy or other social disadvantages, they result in improved knowledge, better patient-clinician communication, and reduced decisional conflict [16].

Although reduced grade reading level is a key element of addressing health literacy, other health literacy and accessibility principles should also be considered when designing digital decision aids. In Table 1, we highlight several ways in which evidence-informed guidelines for understandable, actionable, and inclusive health information, as well as decision aid development guidelines, can intersect and be applied for more equitable digital decision aids. Our list is not exhaustive, but we hope it will serve as a starting point for researchers and other developers of digital decision aids to attend to the additional and often ignored needs of underrepresented groups. As we elaborate below, transparent, detailed reporting of strategies adopted to meet the needs of users with lower health literacy should be prioritized in publications to enhance the evidence base.

**Table 1.** Guidelines and tools to enable more inclusive digital decision aid design and implementation.

Tool	Details^a^	Example applications^a^ to digital decision aids
Patient Education Materials Assessment Tool (PEMAT) [25]	Items to assess understandability and actionability of patient education materials	Break or “chunk“ information into short sections: Using drop-down lists with more detailed information for users who desire it Include informative headings (ie, a disclosure) so users can determine whether information is relevant and allow for a trigger button to easily expand or collapse Define medical terms when they are used: Use popovers that remind users of medical term definitions, if desired Ensure that an informative label is provided, and that it is easy to close (using a large, visible close button or a keyboard shortcut to dismiss) Ensure the material allows users to hear material clearly: Provide narrations of key information in multiple languages for the target users
Web Content Accessibility Guidelines (WCAG; currently being updated) [26]	Standards to ensure people with a wide range of disabilities can perceive, understand, and navigate web content	Perceivable (text alternatives, time-based media, and adaptability): provide section for transcripts of nontext content (eg, icon arrays and images) so users can change forms (eg, large print, braille, speech, and symbols), provide captions for any prerecorded audio-content, and avoid flashing lights, moving images, and animation or automatic slideshows Operable (keyboard accessibility and navigability): ensure all navigation is operable through a keyboard interface, include a title to describe topic or purpose of each page and element of decision aid, and ensure all buttons labeled appropriately and informatively (eg, not “click here”) Understandable (input assistance): provide labels or instructions if including any interactive elements that require user to input content Robust (compatibility): include in testing process whether the tool is compatible with assistive technologies
IPDAS^b^ [27], SUNDAE^c^ [28] checklists, and STROBE^d^-Equity extension [29]	Reporting checklists for decision aid development and evaluation strategies	Use checklists in development and evaluation studies with particular attention to advancing evidence base for underrepresented end user groups
Sydney Health Literacy Lab (SHeLL) Editor [30]	Supports timely and scalable uptake of health literacy guidelines	Input any text content being included in decision aids to receive real-time feedback on reducing grade reading level, as well as avoiding long sentences, complex words, and passive voice

a List is not exhaustive and provides some examples of applying guidelines to digital decision aids (PEMAT, WCAG, and SHeLL Editor).

b IPDAS: International Patient Decision Aid Standards.

c SUNDAE: Standards for Universal reporting of patient Decision Aid Evaluation.

d STROBE: Strengthening the Reporting of Observational studies in Epidemiology.

### Meaningful Co-Design With Typically Underrepresented End Users

As online access to and integration of digital decision aids in health platforms increases, context-specific co-design that considers the needs of underserved end users is essential. A recent meta-analysis of digital patient decision support tools for atrial fibrillation treatments found most studies reported co-design with consumers, but socioeconomically disadvantaged groups were poorly represented [31] (a common issue in co-design [32]). In the absence of evidence-based guidance for decision aid co-design with underserved end users, we draw on broader co-design equity insights, as well as the experiences of our authorship team with expertise in co-design from both consumer and researcher perspectives. It is vital to ensure the co-design process with representatives of underrepresented groups is proactive and authentic to avoid consumers experiencing a “faux-design” process in which they are being asked to provide thoughts about a solution that has already been decided on.

Meaningful co-design that prioritizes equity means starting at the precommencement stage to understand the perspectives and experiences of those who are particularly at risk of exclusion and inaccessibility when it comes to engaging with such digital tools [33]; seeking their genuine influence in the design and testing process, adjusting methods, and providing resources to support meaningful contributions; and building trusting relationships in the context of power imbalance [34]. Co-design with groups who may be perceived as “vulnerable” likely also requires reframing perspectives to an asset-based position and narrative [35]. We should shift from viewing lived experiences as “problems” or “deficiencies” to instead viewing them as ways of engaging with “invisible communities,” by recognizing and reinforcing their agency, choice, and power throughout the co-design process [36]. These processes can take more resources and time, but we need to take steps toward ensuring that those already experiencing the harms of inequities in the health system have equal opportunity to not only access digital decision aids but also influence their design for optimal use among underrepresented groups.

Using an implementation framework as early as possible in the co-design process that explicitly considers preimplementation planning [37] (eg, the preparation phase of the EPIS [exploration, preparation, implementation, sustainment] framework [38]) would be particularly useful for guiding a structured clarification of the needs of all stakeholders (ie, consumers, clinicians, and health services or organizations). Different needs for support unique to particular user groups can be mapped and prioritized, and then strategies can be explored to address such needs in earlier stages with the view of eventual implementation. For example, a well-documented facilitator for shared decision-making is consumer preparation for an encounter, so users who are typically underserved in our research should be consulted prior to the implementation phase to clarify whether and how the design of a digital decision aid could facilitate this preparatory process. Although we focus on resource design recommendations here, support beyond the decision aid itself may be needed, especially for users with multiple layers of disadvantage, such as leveraging specific in-community support. Mapping needs for specific groups of clinicians may also reveal supplementary strategies needed to enhance equitable uptake (eg, shared decision-making training programs that integrate health literacy [39]). Considering the exponential growth in availability of decision support resources online, strategies are likely needed to support clinicians to find the most appropriate tool to endorse for individual health consumers with specific needs. Supporting such streamlined access to health-literate, evidence-based tools is possible through early consultation with clinicians and health services (eg, integration of digital decision aids with existing clinical workflows and software [23,40]).

### Generating Evidence on Interactivity, Tailoring, and AI

While evidence is still emerging in this space, if leveraged appropriately, there is likely value in utilizing emerging technologies in decision aid development relating to interactivity, tailoring, and AI. We are likely underdeveloping existing digital decision aids with regard to enhancing features for diverse ranges of users.

#### Interactivity

Interactivity in digital decision aids means a user is not only reading with one-way engagement but rather given control over various features. This might include deciding which content is viewed, the way numeric risk information is visualized, or completing exercises to clarify what matters most to them. Interactivity has been positively perceived in qualitative studies developing digital decision aids for users with lower health literacy, especially for the process of values clarification in which a user can receive feedback to come to a decision that aligns with their personal values [24,41]. For an example of an interactive digital decision aid, researchers used a community-engaged approach to develop a multimedia website about precision oncology and clinical trial participation for African American patients. Interactive features available included a quiz for users to check their understanding while progressing through the material and a chatbot, “LEIA” (Leading Equity Increasing Accessibility), to help users navigate the website or direct them to useful support resources [42]. However, there is limited evidence on the effectiveness of different types of interactivity for presenting numeric information in decision aids [43], so its potential role for users already at a disadvantage when navigating our health systems should be further explored. Positive perceptions are not sufficient for drawing conclusions about effectiveness and supporting implementation. Therefore, we need more evidence on how different types of interactive features influence knowledge for users with lower health literacy, as well as whether effects differ across clinical and user contexts. Adverse impacts are possible, such as distracting users from understanding relevant statistical information [44]. Ensuring those with lower health literacy experience the intended benefits of interactivity in digital decision aids is key [45].

#### Tailoring

Digital features could also allow for more individual tailoring of decision aid content [46]. First, tailored information could be presented by allowing consumers to enter their own preferences for more or less detailed information, allowing for greater acceptability and understanding. Although benefits of self-tailoring information in digital decision aids are not well understood, broader health communication evidence suggests increased engagement, more in-depth processing of information, greater recall, and greater intention to engage in the related health behavior change [47].

More personalized risk information tailored according to individual attributes (eg, age, family history, and ethnicity) is also possible, which would not only provide more accuracy but also better suit user preferences, including those with lower health literacy [45]. Concerns from consumers and clinicians have previously been documented that personalized risk estimates could be disregarded due to perceived or actual inaccuracies [48,49], but current evidence is needed in the context of an increasingly digitalized health system, as these reviews were conducted over a decade ago. For example, more recently, health professionals reported that decision aids could be better implemented if they were autopopulated from risk assessments from medical record data via clinical audit software, allowing consumers and clinicians to readily discuss the latest evidence tailored to individual risk [50]. Further research is required to elucidate the potential benefits and risks of these types of tailoring and to establish how best to communicate risk in a way that builds consumer and clinician trust.

#### AI

Finally, AI-enabled decision aids are increasingly being developed, but the way this technology can be integrated is rapidly evolving, with limited evidence to guide equitable design and implementation. Concerns about bias and exacerbated health inequities were highlighted in a systematic review of both consumer and clinician perspectives [51], but this was regarding the use of AI to tailor risk information using sophisticated models. Since the launch of ChatGPT in November 2022, public perceptions toward AI have evolved and there has been a rapid increase in consumer access, including its use for health information among those with lower health literacy [52]. We are at a real inflection point for application of large language models (a subset of generative AI trained on extensive datasets) in health communication [53]. Given consumers already use generative AI to access health information, with those who face barriers to health care access even more likely to [52], we must ensure its beneficial applications are pursued wherever possible, such as in enhancing digital decision aids for underserved groups.

Potential benefits that come with rapid improvements in large language models could be particularly useful for addressing some aspects of inequity in health information. Generative AI can reduce the grade reading level of health education materials [54], as well as support question asking and reflective processes for values clarification through greater interactivity and opportunity to engage without time pressure or shame. Although there can be occasional inaccuracies when it is used to simplify health information with appropriate prompts [54], generative AI is continually evolving, including some recent improvements in accuracy when used for health care in newer versions. A retrieval-augmented generation framework can also address limitations by allowing health information developers to control the quality of the information its output is based on [55]. AI may also be useful for overcoming a research team’s bias, with some evidence demonstrating that generative AI was able to identify a broader range of options and benefits and harms, promoting a more holistic perspective that caters to diverse users with different values [56]. Generative AI also allows for scalable and affordable translation of digital decision aid content into multiple languages. There are practical risks of overreliance on AI, especially for translating rarer or emerging languages in multicultural societies [57], so co-design and technology improvements are essential. However, we should not avoid leveraging the potential benefits of addressing inequities in digital decision aids, especially if the alternative (not using AI) results in continued proliferation of digital decision aids that only suit the needs of people with higher health literacy, those who are highly educated, and other socially advantaged groups, often due to resource constraints.

## Prioritizing Equity in Digital Decision Aid Environments and Implementation

National health systems recommending consumer engagement in health standards and clinical guidelines should further bolster the above design and research recommendations by investing in digitally enabled shared decision-making environments that are accessible and inclusive. To address key actions from the World Health Organization Global Patient Safety Action Plan 2021‐2030 [58], we call for policymakers to support researcher and organization initiatives by investing at a national level in (1) a central, standardized portal for both developers and users of digital decision aids with practical resources and methods embedded for equitable design and (2) digital inclusion initiatives to ensure more equitable access to consumer-facing digital decision support resources.

### National Portals

Central, national portals that standardize consumer access to meaningfully co-designed digital decision aids that meet the needs of users with varying levels of health literacy should be a key investment for governments endorsing consumer and patient engagement in health care decisions. Sustained uptake of shared decision-making is currently hindered by inefficiency due to duplication of efforts, lack of standardization in resource access, and limited measurement and monitoring through feedback and reporting [59]. For example, implementation remains unlikely in contexts where uptake is driven by individuals or teams and funded by fixed-term, health context–specific research grants. Nationally governed portals could also allow for regular updates as evidence-based innovations emerge (eg. in interactivity, tailoring, and AI). Although existing portals in some countries have increased availability and access to digital decision aids (eg, Denmark), provisions to integrate digital features that prove effective in supporting health decisions among priority groups should also be prioritized [14]. Finally, a portal could of course expand beyond just digital decision aids, supporting standardized access to many forms of decision support and communication tools (eg, question prompt lists) as well as access for researchers and other developers to templates and design resources.

National initiatives to integrate standardized decision aid portals with information technology systems already used in clinical workflows could also advance efforts for more sustained, equitable shared decision-making. Integration could support continued monitoring and feedback of patient-reported outcomes in routine clinical practice. Ideally, such feedback would improve clinical attitudes toward supporting those with lower health literacy in shared decision-making [60], while also facilitating access to tools for those who need more support to use them. However, such integration is not easy or fast, requiring substantial time and significant institutional support. The integration of Option Grid decision aids in 5 medical centers in the United States took 18 months, with facilitators including clinical champions advocating at an institutional level, guidance from an experienced software technologist, the use of an emerging industry standard app platform, and the willingness of the software development team [61]. Particularly if integrating AI-enabled decision aids into the clinical setting that simplify information and support question asking, more evidence would also be needed for building trust among consumers and clinicians.

### Digital Inclusion Initiatives

Even if optimally designed and implemented to meet the needs of diverse consumers and clinicians, the digital decision aid environment must also be addressed in terms of digital inclusion. We urge policymakers to begin or continue pursuing digital inclusion by supporting initiatives to address access, affordability, and digital ability, while ensuring good monitoring for national improvements (eg, the Australian Digital Inclusion Index) [62]. Such investments will increase the potential for digitally enabled patient engagement and shared decision-making to become more responsive, sustainable, and equitable in the next decade (as per the goals cited in many national digital health policies). In the meantime, researchers, health services, and organizations developing digital decision aids should consider dissemination strategies that ensure printable formats that meet health literacy principles are available alongside digital versions.

## Conclusions

Shared decision-making research and practice can leverage exciting digital health system capabilities to advance implementation efforts. However, evidence-based strategies need to be prioritized by researchers and policymakers to ensure equitable design and implementation by following health literacy and accessibility guidelines, meaningfully co-designing tools in partnership with underrepresented consumers, health care providers, and health services, and generating evidence among underrepresented groups on the impacts of innovations such as interactivity, tailoring, and AI on decision outcomes. The promise of better-quality care through tools to support patient engagement can be achieved for all if those involved in design proactively prioritize the time and resources needed to meet diverse end user needs. However, policymakers must also ensure centralized availability and access to such tools, while also prioritizing country-specific digital inclusion initiatives.

## Abbreviations

EPIS: Exploration, Preparation, Implementation, Sustainment
LEIA: Leading Equity Increasing Accessibility
NICE: National Institute for Health and Care Excellence
